# Rotational Atherectomy to Treat in-Stent Recurrent Protruding Calcified Nodule

**DOI:** 10.1016/j.jaccas.2024.102815

**Published:** 2025-01-15

**Authors:** Saad M. Ezad, Joshua J. Hon, Divaka Perera, Kalpa De Silva

**Affiliations:** a Cardiovascular Division, St Thomas' Hospital, Guy's and St Thomas' NHS Foundation Trust, London, United Kingdom; b British Heart Foundation Centre of Research Excellence at the School of Cardiovascular and Metabolic Medicine and Sciences, King’s College London, London, United Kingdom; c Faculty of Medicine, Imperial College London, London, United Kingdom

**Keywords:** calcific nodule, in-stent re-stenosis, OCT, rotablation

## Abstract

A 73-year-old man presented with acute coronary syndrome secondary to stent failure. Intravascular imaging identified a recurrent protruding calcific nodule as the mechanism, which was effectively treated with low-speed rotablation, resulting in ablation of the nodule allowing the application of a drug-coated balloon.

## Graphical Abstract

**Figure:**
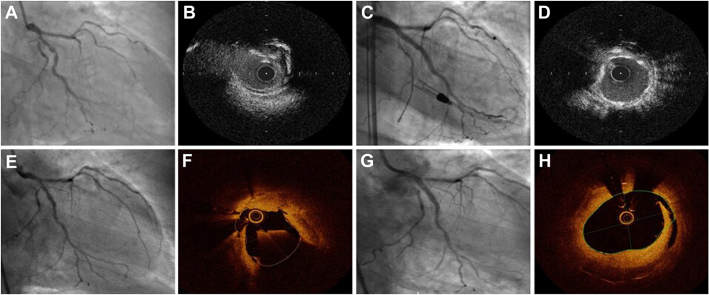

## History of presentation

A 73-year-old gentleman presented with progressive shortness of breath and chest pain to his local district general hospital. Clinical examination was consistent with decompensated heart failure evidenced by peripheral edema, rales to the mid zones and elevated jugular venous pressure; an apical pan-systolic murmur consistent with mitral insufficiency was also noted. Initial investigations revealed a new anemia with a hemoglobin of 8.5 g/dL (12.5-17.0 g/dL), elevated high-sensitivity troponin I of 1,085 ng/L (<16 ng/L) without dynamic change when repeated (3-hour troponin 1,070 ng/L), whereas electrocardiogram demonstrated sinus rhythm with a known left bundle brunch block. The patient was stabilized with diuresis and medical therapy whilst further investigations were undertaken.

**Take-Home Messages**

• This case highlights the importance of intravascular imaging in identifying the mechanism of stent failure.
• Low-speed rotational atherectomy can effectively ablate recurrent in-stent calcific nodules.

## Past Medical History

**Figure 1:**
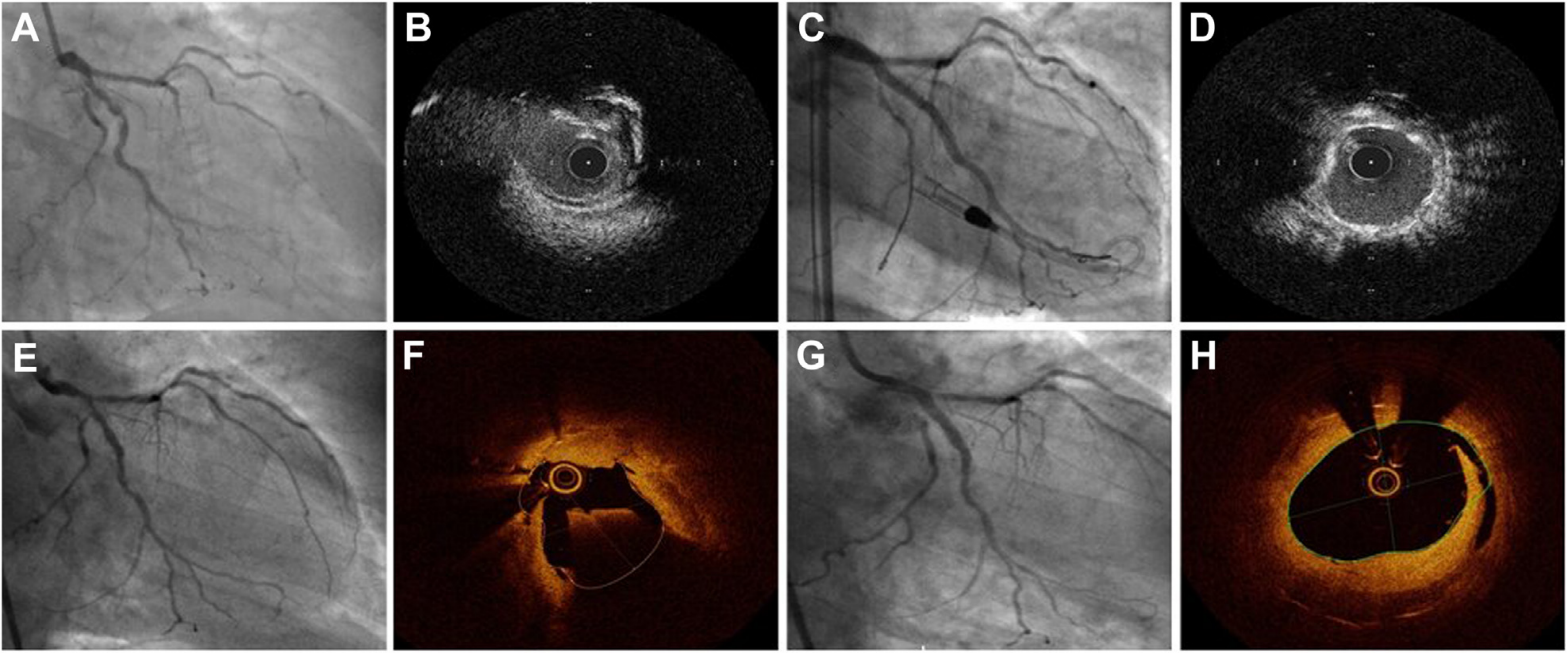
Coronary Angiography and Intravascular Imaging (A) Baseline coronary angiography showing severe calcific disease in the distal left main stem and proximal left circumflex. (B) Intravascular ultrasound image demonstrating protruding calcific nodule at the ostium of the left circumflex. (C) Postintervention coronary angiography. (D) Postintervention intravascular ultrasound of well-expanded stent at the site of the nodule. (E) Coronary angiography following presentation with recurrent acute coronary syndrome. (F) Optical coherence topography following predilation demonstrates calcific nodule protruding through the stent in the distal left main stem. (G) Coronary angiogram following rotational atherectomy and treatment with drug-coated balloon. (H) Postintervention OCT showing successful ablation of the nodule and dissection within the neointimal tissue.

He had presented 15 months previously with a non–ST-segment elevation myocardial infarction, coronary angiography at the time revealed chronic total occlusions of his left anterior descending and right coronary arteries. The left main stem (LMS) and left circumflex (LCx) arteries had severe calcific stenosis (Figure 1A, Video 1). Cardiac magnetic resonance imaging confirmed non-viable left anterior descending coronary artery and right coronary artery territories and an ejection fraction of 32%. Following heart-team discussion he underwent percutaneous coronary intervention to his LMS and LCx with Impella (Abiomed) support. Intravascular ultrasound showed nodular calcification at the ostial LCx (Figure 1B, Video 2) which was modified with intravascular lithotripsy. A 3.0-mm drug-eluting stent was placed in the mid to distal LCx and overlapped proximally with a 3.5-mm drug-eluting stent in a provisional manner to the LMS ostium (Figure 1C). Proximal optimization technique was performed with a 5.0-mm NC balloon (Video 3). Intravascular ultrasound confirmed an eccentric but well expanded stent with a minimal stent area (MSA) of 7.4 mm^2^ in the ostial LCx (Figure 1D, Video 4).

## Differential diagnosis

The patient had several possible explanations for decompensating including anemia, valvular heart disease, and acute coronary syndrome.

## Investigations

Transthoracic echocardiography revealed an ejection fraction of 21% in keeping with his known ischemic cardiomyopathy. Moderate mitral regurgitation was present similar in appearance to his previous echocardiogram 15 months previously. Given the complexity of coronary disease and presentation with decompensated heart failure noninvasive investigation of the coronary anatomy was undertaken with computed topography coronary angiography, which suggested significant restenosis within the previously implanted LCx stents. Esophago-gastro-duodenoscopy and colonoscopy were felt to be high risk in the presence of a possible acute cardiac event, following discussion the patient was therefore transfused 2 units of packed red blood cells and transferred to our tertiary institution for invasive angiography and further management.

## Management

Coronary angiography confirmed severe in-stent restenosis throughout the LCx and LMS (Figure 1E, Video 5). Following initial predilatation with a cutting balloon, optical coherence topography (OCT) showed development of a new protruding calcific nodule, deforming stent struts in the distal LMS (Figure 1F, Video 6). The MSA was 2.94 mm^2^, rotational atherectomy (RA) was performed with a 1.75-mm burr initially at 200,000 RPM followed by sequential reduction in burr speed to 140,000 RPM to maximize luminal gain.^1^^,^^2^ Repeat OCT showed the nodule had been significantly debulked with almost complete ablation. Prolonged balloon inflations were poorly tolerated; however, given the modification of the calcific nodule and acceptable minimal lumen area on OCT a drug-coated balloon was applied. The need for prolonged inflation (60 seconds) of the drug-coated balloon resulted in hypotension, which resolved with bolus inotropic support. A final MSA in the LMS of 9.3 mm^2^ was achieved (Figures 1G and 1H, Videos 7 and 8). Following optimization of medical therapy for heart failure, the patient was discharged home with a plan for outpatient implantable cardioverter-defibrillator insertion.

## Discussion

To the best of our knowledge, this is the first description of an in-stent protruding calcific nodule treated with reduced speed RA and highlights the importance of utilizing intravascular imaging to understand the cause of stent failure allowing targeted intervention. Calcific nodules are increasingly being recognized as a cause of acute coronary syndromes and stent failure, in particular with eruptive calcified nodules although in the present case the nodule appeared noneruptive.^3^ At the time of the index intervention, the nodule was adequately modified by intravascular lithotripsy, which has recently been shown to be safe and effective.^4^ For the current presentation, RA was chosen so as to cause less hemodynamic perturbance than may have been observed if intravascular lithotripsy had been chosen owing to the need for prolonged balloon inflation to deliver therapy.

RA was initially undertaken at 200,000 RPM, which is higher than traditionally used to reduce the risk of burr entrapment within the previously implanted stent and the speed was only reduced once the burr passed freely through the lesion. Although the wire bias was not favorable, we believe the use of reduced speed RA allowed modification of the nodule as this has been shown to increase the achieved minimal luminal area as compared with high-speed RA independent of the burr size.^1^^,^^2^

Intravascular lithotripsy can also effectively modify calcific nodules with luminal gain comparable to that seen with non-nodular calcium, however, eruptive nodules demonstrated greater deformability as compared with noneruptive nodules.^5^ Therefore, in cases of noneruptive nodules such as in the current report atherectomy techniques may be more effective at modifying the nodule. Laser atherectomy can be considered in cases of device uncrossable lesions, which can then facilitate synergistic use of other calcium-modifying devices. Orbital atherectomy may also have achieved similar results as the eccentric orbit of the burr is increased at lower atherectomy speeds, which could therefore have modified the nodule despite unfavorable wire bias. In this case, however, the use of orbital atherectomy was contraindicated due to the risk of burr entanglement in the previously implanted stent.

Calcific nodules are associated with an increased risk of major adverse cardiac events, recurrent acute coronary syndrome and target lesion revascularization with over 80% of stent failure in such cases due to re-emergence of the nodule within the stent.^6^ The present case describes 1 potential strategy for treating such presentations, however, further research is needed to identify the optimal management strategy and ideally strategies to prevent re-emergence of the nodule.

## Follow-up

The patient was reviewed 6 months postdischarge and reported no symptoms of angina or heart failure.

## Conclusions

Intravascular imaging is essential to understand the cause of stent failure, once a calcific nodule has been identified multiple ablative therapies can be applied depending on the patient characteristics and clinical scenario. In the present case, low-speed RA achieved excellent luminal gain allowing application of a drug-coated balloon and avoiding a second layer of stent.

## Funding Support and Author Disclosures

Dr Ezad is supported by a British Heart Foundation Clinical Research Training Fellowship (FS/CRTF/21/24118). The authors have reported that they have no relationships relevant to the contents of this paper to disclose.

## Appendix

Video 1 Baseline coronary angiography showing severe calcific disease in the distal left main stem and proximal left circumflex.

Video 2 IVUS image demonstrating protruding calcific nodule at the ostium of the left circumflex.

Video 3 Post intervention coronary angiography showing a good result following intervention in the left main stem and left circumflex.

Video 4 Post intervention IVUS demonstrating well expanded stents at the site of the nodule and in the left main stem.

Video 5 Coronary angiography following presentation with recurrent acute coronary syndrome showing severe in-stent re-stenosis.

Video 6 OCT following pre-dilation demonstrates calcific nodule protruding through the stent in the distal left main stem.

Video 7 Coronary angiogram following rotational atherectomy and treatment with drug coated balloon.

Video 8 Post intervention OCT showing successful ablation of the nodule and dissection within the neointimal tissue with acceptable luminal gain.
